# Evaluation of a New Glycomacropeptide-Based Protein Substitute in Powdered and Liquid Format in Patients with PKU

**DOI:** 10.3390/nu15163580

**Published:** 2023-08-14

**Authors:** Marta Delsoglio, Rebecca Capener, Anita MacDonald, Anne Daly, Catherine Ashmore, Charlotte Ellerton, Sarah Donald, Lisa Gaff, Louise VanDorp, Rachel Skeath, Camille Newby, Georgina Dunning, Clare Dale, Inderdip Hunjan, Lucy White, Heather Allen, Gary P. Hubbard, Rebecca J. Stratton

**Affiliations:** 1 Research and Innovation, Nutricia Ltd., White Horse Business Park, Trowbridge BA14 0XQ, UK; 2 Dietetic Department, Birmingham Children’s Hospital, Birmingham B4 6NH, UK; 3 University College London Hospitals NHS Foundation Trust, London WC1N 3BG, UK; 4 Cambridge University Hospitals NHS Foundation Trust, Cambridge CB2 0QQ, UK; 5 Great Ormond Street Hospital for Children NHS Foundation Trust, London WC1N 3JH, UK; 6 Bristol University Hospitals NHS Foundation Trust, Bristol BS1 3NU, UK; 7 Queen Elizabeth Hospital, Birmingham B15 2TH, UK; 8 Bradford Teaching Hospitals NHS Foundation Trust, Bradford BD5 0NA, UK; 9 Sheffield Children’s NHS Foundation Trust, Sheffield S10 2TH, UK; 10 Faculty of Medicine, University of Southampton, Southampton SO16 6YD, UK

**Keywords:** phenylketonuria, PKU, glycomacropeptide, protein substitute

## Abstract

(1) Background: Good adherence to a Phe-restricted diet supplemented with an adequate amount of a protein substitute (PS) is important for good clinical outcomes in PKU. Glycomacropeptide (cGMP)-PSs are innovative, palatable alternatives to amino acid-based PSs (AA-PS). This study aimed to evaluate a new cGMP-PS in liquid and powder formats in PKU. (2) Methods: Children and adults with PKU recruited from eight centres were prescribed at least one serving/day of cGMP-PS for 7–28 days. Adherence, acceptability, and gastrointestinal tolerance were recorded at baseline and the end of the intervention. The blood Phe levels reported as part of routine care during the intervention were recorded. (3) Results: In total, 23 patients (powder group, *n* = 13; liquid group, *n* = 10) completed the study. The majority assessed the products to be palatable (77% of powder group; 100% of liquid group) and well tolerated; the adherence to the product prescription was good. A total of 14 patients provided blood Phe results during the intervention, which were within the target therapeutic range for most patients (*n* = 11) at baseline and during the intervention. (4) Conclusions: These new cGMP-PSs were well accepted and tolerated, and their use did not adversely affect blood Phe control.

## 1. Introduction

Phenylalanine hydroxylase deficiency or phenylketonuria (PKU) is a rare, autosomal recessive disorder characterised by an inability to metabolise the essential dietary amino acid phenylalanine (Phe) into tyrosine (Tyr), due to a mutation in the genes that code for the phenylalanine hydroxylase (PAH) enzyme [1]. Its prevalence varies worldwide, with an average of about 1:10,000 newborns in white or east-Asian populations [2]. If untreated without appropriate dietary management from birth, PKU leads to increased blood and brain phenylalanine (Phe) levels, tyrosine deficiency, and irreversible neurological damage [3]. Elevated Phe levels, especially at critical times of growth and development, can result in permanent damage to the brain, significant intellectual disability, and reductions in IQ [4]. Clinically, untreated patients may also develop severe epilepsy and behavioural, psychiatric, and movement problems, as well as a light pigmentation of the skin, eyes, and hair, eczema, and a musty odour [2]. However, with strict dietary management [5,6,7,8], patients with PKU can have reasonably normal cognitive development [9,10]. Two pharmaceutical treatments are also currently available for some patients: tetrahydrobiopterin (BH4) for BH4-responsive patients [11] and pegylated phenylalanine ammonia lyase for adult patients (>16 years in Europe), which decrease blood Phe concentrations and improve dietary Phe tolerance [12,13,14]. Sepiapterin, a natural precursor of intracellular BH4, has also been shown to lower blood Phe concentrations and is currently under clinical development [15]. Additional therapies targeting Phe in the gastrointestinal tract are under investigation, such as engineered *Escherichia coli* Nissle, which expresses Phe ammonia lyase and other Phe-degrading enzymes [16], or inhibitors of the major intestinal absorption pathway for phenylalanine, the epithelial apical membrane amino acid transporter B0AT1 (slc6a19), showing the potentiality of B0AT1 as a target for treating PKU, among other conditions [17,18,19].

Global expert guidelines recommend the early and lifelong management of patients with PKU [20,21]. The primary goal of this management is to maintain the blood Phe levels within acceptable ranges in order to achieve normal growth and neurological outcomes. The cornerstone of achieving this is a (severely) restricted natural protein diet supplemented with a low or Phe-free protein substitute (PS) and permitted low-protein foods (e.g., some vegetables, fruits, and special low-protein foods) to meet macro- and micronutrient requirements [22,23,24]. Most of the protein in a Phe-restricted diet is in the form of a PS, which typically provides around 80% of the total protein intake in classical PKU [25]. Due to the strict nature of this diet (e.g., no meat, fish, dairy products, wheat flour, or bread), micronutrient deficiencies may occur, such as vitamins (e.g., B12), minerals (calcium, selenium, and zinc), and essential fatty acids (DHA) [26,27,28,29,30,31,32]. This highlights the importance of a fortified PS, not only for meeting protein needs, but also as a major supply of these other critical nutrients. Adherence to this diet and PS is a key determinant of good clinical outcomes, including metabolic stability, appropriate growth, a good micronutrient status, and normal neurological/psychosocial function [5,33].

Phe-free PSs used for the management of PKU are traditionally based on synthetic amino acids (AAs), and these have a characteristic strong odour and bitter taste, which can affect dietary adherence and outcomes in the longer term [34]. It is common for dietary adherence to worsen as a child gets older, with patients commonly relaxing or abandoning their diet during adolescence and adulthood [23,34,35,36,37]. In the last decade, PSs based on casein glycomacropeptide (cGMP), which have a more neutral taste, have been introduced [38]. cGMP is a widely used food ingredient that is naturally very low in Phe. Because of its very low levels of other essential amino acids (arginine, cystine, histidine, leucine, tyrosine, tryptophan, and valine), it is supplemented with these AAs to produce a high-biological-value PS [39,40]. Studies have reported that cGMP-PSs provide a palatable, alternative source of protein that may improve the adherence for patients with PKU [38,39,41,42,43], with cautious monitoring required for children with classical PKU due to a possible increase in their blood Phe concentrations [44]

The dietary restrictions required for patients and their families are challenging, and newer variants of PSs that are clinically efficacious and widen the variety and choice of the PSs available may promote improved dietary adherence [35,45]. The objective of this study was to investigate the adherence, acceptability, gastrointestinal (GI) tolerance, and metabolic control achieved with a new cGMP-based PS (cGMP-PS) in powder and liquid formats in children and adults with PKU.

## 2. Materials and Methods

### 2.1. Study Design

This was a prospective, multi-centre intervention study aimed at evaluating a new cGMP-PS in liquid and powder formats in children and adults with PKU. Potentially eligible subjects from eight UK metabolic centres (Cambridge University Hospitals NHS Foundation Trust (Addenbrookes); University College London Hospitals NHS Foundation Trust; Birmingham Children’s Hospital NHS Foundation Trust; Queen Elizabeth Hospital Birmingham NHS Foundation Trust; Bristol Royal Hospital for Children; Great Ormond Street Hospital, Sheffield Children’s NHS Foundation Trust, and Bradford Teaching Hospital NHS Trust) who met the entry criteria were invited to participate. The inclusion criteria were: male or female, over 3 years of age, diagnosed with classical or moderate PKU, adherent with a current PS that provides >10 g of protein equivalent/day for at least 1 month prior to the trial commencement, prescribed a daily restricted Phe allowance, and willing to provide informed consent. The exclusion criteria were: pregnant or lactating, requiring parenteral nutrition, major hepatic or renal dysfunction, participation in other studies within 1 month prior to entry into this study, allergy to any of the study product ingredients, including milk, and investigator concern around willingness/ability to comply with the protocol requirements.

To participate in the study, the patients were required to replace their current PS wholly or partially with at least one serving/d (providing 20 g of protein equivalent/day) of the study products for 7–28 days. The dose was specified on an individual basis by the dietitian responsible for the patient’s nutritional management. The study protocol was approved by the South West—Central Bristol Research Ethics Committee and registered at clinicaltrials.gov (NCT05062226). UK Health Research Authority (HRA) approval and local NHS R&D/site approval was obtained from all the sites involved. The study was conducted in accordance with the Declaration of Helsinki and Good Clinical Practice (GCP) guidelines. All the patients or patients’ parents provided written informed consent before any study-related procedures were performed.

### 2.2. Outcomes Measures

Information on the acceptability and GI tolerance of the patients’ current PS and the new powdered and liquid cGMP-PS was collected at baseline and at the study end, respectively, via a standardised questionnaire. The acceptability parameters were recorded on a Likert scale (ratings: ‘Great’, ‘Good’, ‘Ok’, ‘Bad’, and ‘Terrible’) for appearance, taste, smell, texture, ease of mixing (for the powder cGMP-PS only), ease of taking, aftertaste, breath smell, and overall acceptability. The GI tolerance parameters (ratings: ‘none’, ‘mild’, ‘moderate’, and ‘severe’) were diarrhoea, constipation, nausea, vomiting, abdominal discomfort, bloating, flatulence, and burping. Adherence to the recommended intake of the study product was assessed by the managing dietitian.

At baseline, the height, weight, and blood Phe results closest to the time of recruitment were recorded. Any blood Phe results that were reported as part of routine clinical management during the intervention period were also recorded.

### 2.3. Study Products

Both study products (Nutricia Ltd., Trowbridge, UK) were cGMP-PSs supplemented with vitamins, minerals, and trace elements. A single serving of each product (a 33.4 g sachet of the powdered product reconstituted with 180 mL of water or a 250 mL carton of the liquid product) provided a 20 g low-phe protein equivalent and contained 36 mg of phe/20 g of a protein equivalent (see Appendix A Table A1 for full nutritional profile). The protein equivalent sources in both product formats were: casein glycomacropeptide isolate (from cow’s milk) supplemented with additional l-Leucine, l-Tyrosine, l-Arginine, l-Histidine, l-Valine, l-Cystine, and l-Tryptophan to compensate for the low levels of these AAs in cGMP. Both products had the same AA profile, which was designed to have an essential AA profile that reflected that of a high-biological-value protein. The vitamin, mineral, and trace element profiles of the study products were closely aligned on a per 20 g protein equivalent basis.

### 2.4. Data Analysis

Descriptive statistics are presented on the characteristics of the study population and the findings for the adherence, acceptability, and GI tolerance of all the patients. The changes in the blood Phe levels between the baseline and study end, where available, were compared for each subject and with their age-specific target treatment ranges. Patients who consumed the study products for <7 days were excluded from the final analysis.

## 3. Results

### 3.1. Baseline Characteristics

The baseline characteristics of the study population are summarized in Table 1. There were 13 patients in the powdered cGMP-PS group and 10 patients in the liquid cGMP-PS group. Both children and adults were included in the study, with a greater proportion of children and adolescents (three children, three adolescents, and four adults) in the liquid cGMP-PS group than in the powder cGMP-PS group (one child and twelve adults). In the powdered study product group, the mean age was 37 years old, with an age range of 12–65 and seven females. In the liquid study product group, the mean age was 24 years old, with an age range of 7–49 and four females. At baseline, all the patients were already prescribed protein substitutes for the dietary management of their PKU, with most patients (77% in the powdered study product group and 60% in the liquid study product group) taking a cGMP-PS as their sole PS or in combination with an AA-PS. The baseline PS provided 61 g of PE/day (SD 15, range 40–80) in the powdered study group and 65 g of PE/day (SD 9, range 60–80) in the liquid study group. The cGMP-PSs included PKU Sphere 20 (Vitaflo), PKU Sphere 15 (Vitaflo), PKU GMPro LQ (Nutricia), and PKU GMPower (Mevalia). The AA-PSs included PKU Air 20 (Vitaflo), PKU Air 15 (Vitaflo), Phlexy 10 tablets (Nutricia), PKU Cooler 20 (Vitaflo), PKU Cooler 15 (Vitaflo), and PKU Lophlex LQ 20 (Nutricia).

### 3.2. Product Intake and Adherence

Twenty patients replaced part of their baseline PS with the study products and three patients took the study products in addition to their baseline PS to better meet their protein requirements. None of the patients took the study products as their sole PS. Most patients (17/23) were prescribed one daily dose of the study products, 5 patients were prescribed two daily doses, and 1 patient was prescribed three daily doses. The mean intake during the study period was 29.2 g of protein equivalent (SD 13.2, range 20–60)), with the majority (17 of 23 patients) taking the study products in combination with other cGMP-PSs (Table 2). None of the patients took the study products as their sole PS.

The total protein prescription and contribution from the PS taken during the intervention are summarized in Table 3, with most patients (15 of 23) taking >80% of their protein equivalent prescription from their PS (AA-PS or other cGMP, combined with the study products). During the study period, the powdered and liquid study products provided 40% (SD 22) and 30% (SD 8) of the protein prescribed, respectively. All the patients in the liquid cGMP-PS group took the product for 28 days and were fully adherent to the volume of the study product prescribed. In the powder cGMP-PS group, 9 of 13 patients took the product for 28 days, with the remaining 4 patients taking the product for between 11 and 25 days. Adherence to the powder cGMP-PS (87%, SD 24) was more variable than that of the liquid, with 5 of 13 patients being described as not fully adherent to the amount of powdered cGMP-PS prescribed for the study.

### 3.3. Acceptability of the Study Products

The overall acceptability of the powdered cGMP-PS was rated as satisfactory (‘Ok’ to ‘Great’) by 77% of patients based on all attributes, which included appearance (92%), smell (92%), taste (77%), texture/mouthfeel (75%), ease of mixing (62%), ease of taking (85%), aftertaste (77%), and smell of breath after taking (75%). For those taking the liquid cGMP-PS, all (100%) the patients rated the overall acceptability as satisfactory (‘Ok’ to ‘Great’), based on the same attributes (excluding ease of mixing) for appearance (90%), smell (80%), taste (80%), texture/mouthfeel (100%), ease of taking (90%), aftertaste (89%), and smell of breath after taking (78%). Appendix A Table A2 and Table A3 show the details of the ratings for each attribute.

### 3.4. Gastrointestinal Tolerance

**Figure 1:**
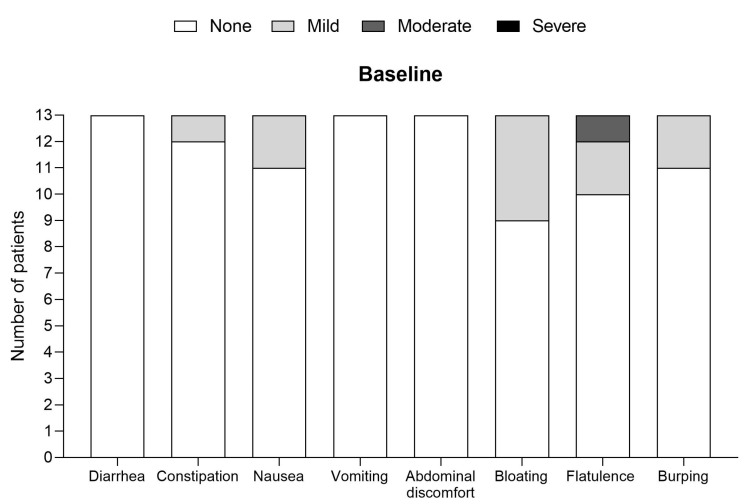

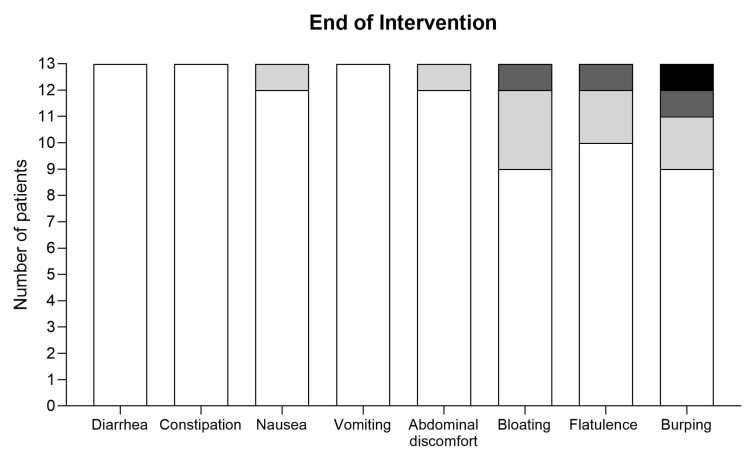
GI symptoms at baseline and while using the new powder cGMP-PS (end of intervention) (*n* = 13).

**Figure 2:**
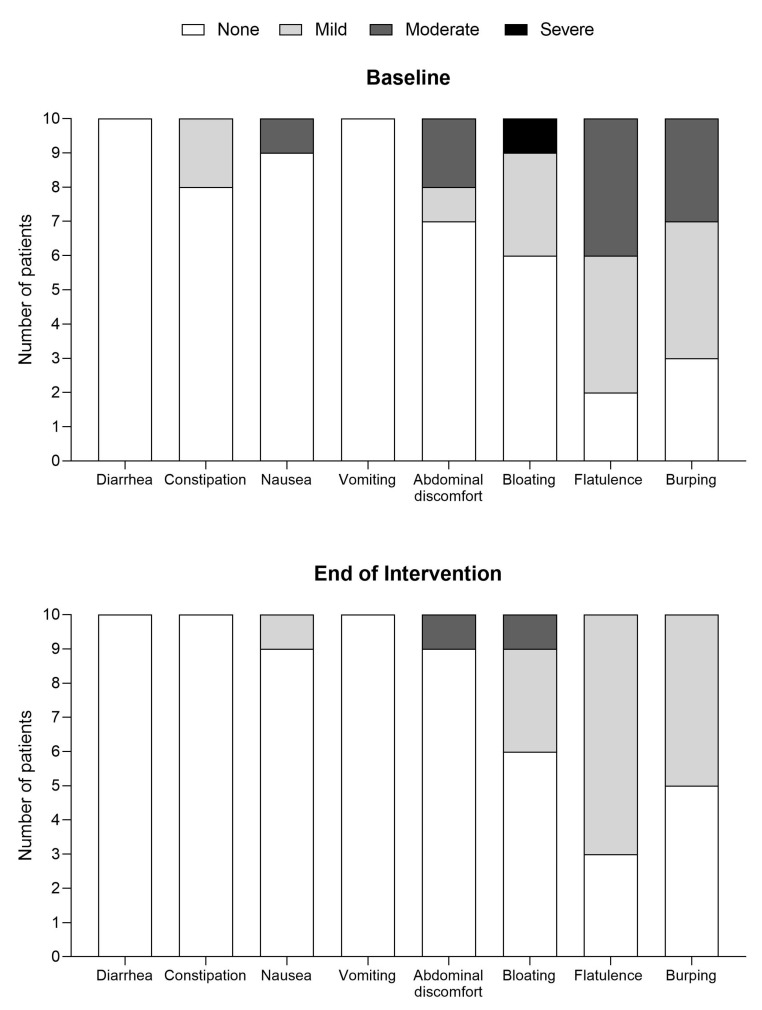
GI symptoms at baseline and while using the new liquid cGMP-PS (end of intervention) (*n* = 10).

Overall, both study products were well tolerated (Figure 1 and Figure 2). There were no reports of diarrhoea, constipation, or vomiting for either study product, and none of the other GI symptoms were recorded as being severe during the intervention period, except for one patient reporting ‘severe burping’ on the powdered cGMP-PS (Figure 1). Mild or moderate bloating, flatulence, and burping were commonly reported GI symptoms at baseline and during the intervention.

Most patients (*n* = 9) taking the powdered study product either had no GI symptoms or no new GI symptoms, with 2 patients reporting an improvement in their constipation (*n* = 1) and nausea (*n* = 1). In total, 2 patients showed new GI symptoms (mild abdominal discomfort and severe burping (*n* = 1), and moderate bloating and moderate burping (*n* = 1) compared to baseline, with both reporting some difficulties in digesting the study product. Most patients taking the liquid cGMP-PS reported improvements in their GI symptoms for the following: flatulence (*n* = 4), burping (*n* = 4), abdominal discomfort (*n* = 2), constipation (*n* = 2), bloating (*n* = 2), and nausea (*n* = 1).

### 3.5. Phe Control

**Figure 3:**
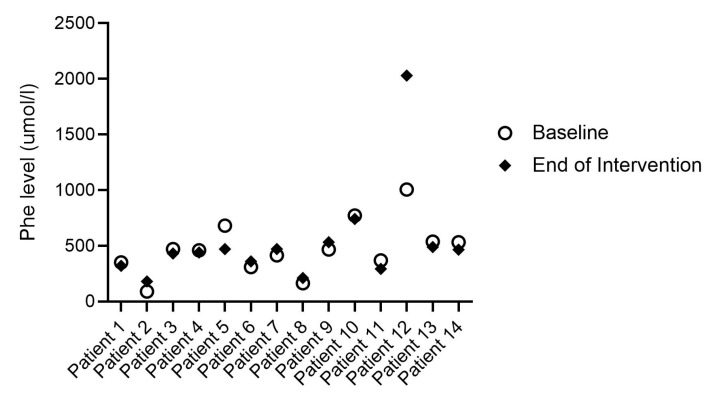
Individual blood Phe levels recorded at baseline and at the end of the intervention (*n* = 14). Patients 1–8 were using the new cGMP-PS powder, while patients 9–14 were using the new cGMP-PS liquid (in combination with other PS for all patients). Patient 12 relaxed her Phe-restricted diet during the study. At baseline, patients 4, 5, 6, 11, and 12 were using solely AA-PS, patients 1, 8, 9, and 10 solely cGMP-PS, and patients 2, 3, 7, 13, and 14 were using a combination of AA-PS and cGMP.

A total of 14 (61%) patients provided Phe measures during the intervention period (6/13 patients in the powder group and 8/10 patients in the liquid group) (Figure 3). Of these, most (*n* = 11, 79%) had blood Phe levels within the target therapeutic range for their age group at baseline and during the intervention. Two adults in the powder group had blood Phe levels above target range on both occasions (771 µmol/L at baseline and 740 µmol/L at end for one subject; 1005 µmol/L at baseline and 2029 µmol/L at end for the other subject). One of these patient’s Phe control worsened during the study period, however, she reported that this was due to a further relaxation of her protein-restricted diet and was not related to the use of the study product. One child in the liquid group had a blood Phe level below target at baseline (90 µmol/L), which increased to within the therapeutic range during the intervention (180 µmol/L). Most of the 14 patients (9/14) combined the study products with another cGMP-PS (*n* = 4) or a combination of another cGMP-PS + AA-PS (*n* = 5). The remaining five patients combined the study products with an AA-PS.

## 4. Discussion

cGMP-PSs are a relatively recent innovation, and these products are increasingly gaining acceptance among patients and healthcare professionals, either wholly or partially replacing patients’ usual AA-PSs. cGMP-PSs have been found to have a taste that is well accepted compared to AA-PSs in studies that have investigated the potential suitability of cGMP-PSs in PKU management [39,43,46,47,48,49]. The new powder and liquid cGMP-PSs investigated in this study were found to be well accepted by the patients, most of whom (17 of 23 patients) had already incorporated a cGMP-PS into their dietary regimen. Although both formats of the study products were rated favourably (77% and 100% for the powder and liquid cGMP-PSs, respectively), the liquid product was rated better than the powdered product. This may have been because the flavour options for the powder product (vanilla and lemonade) were very different to the flavours that the patients were accustomed to, while the neutral flavour of the liquid product was a more readily and widely accepted flavour. The convenience of the presentation of the liquid product as a ready-to-drink product may also have influenced these preferences. Preferences for PSs are highly individual, and patients may be resistant to change, especially when they are established on a preferred product or combination of products. Both study products were well tolerated. In the powder cGMP-PS group, no significant changes in GI symptoms were observed during the intervention period. In the liquid cGMP-PS group, several patients reported improvements in their GI symptoms compared to baseline.

Although the cGMP molecule itself is devoid of Phe, the industrial cGMP ingredient, and therefore all cGMP-PSs, contain a very small amount of Phe, which comes with the extraction process from Phe-containing milk proteins (see Section 2 and Table A1). The residual Phe content of cGMP-PSs may be a limitation for patients with a very low Phe tolerance, such as those with no residual PAH activity or who need to maintain their blood Phe levels within very strict limits. Of the patients who provided information on their Phe levels both at baseline and during the intervention (14 of 23 patients), we did not observe a negative impact on their Phe control, as this did not deteriorate for 13 of the 14 patients. The single patient whose Phe control worsened reported that this was due to a further relaxation of her protein-restricted diet and was not related to the use of the study product. That said, the evaluation of the Phe control in this study should be interpreted with caution given that the intervention period was short, with only 14 patients providing Phe results and the study products providing only a proportion of the total PS intake. It should also be noted that most of these patients (9/14) were already established on a cGMP-PS, either as their only PS or in combination with an AA-PS, and therefore it was not unexpected that their Phe control was unaffected following the introduction of the study product.

Similar observations on the impact of cGMP-PSs on blood Phe control have been made in other studies, most of which have found no significant differences compared to AA-PSs [39,43,46,47,48,49,50], although a few studies have observed that the blood Phe levels increased when patients took a cGMP-PS [51,52]. Due to the natural content of Phe in both study products, the impact on metabolic control may have varied from patient to patient, and it is recommended that caution should be exercised with the use of cGMP-PS in children with classical PKU [40].

Achieving the intake recommendations for protein in PKU can be challenging for patients, as most of these protein recommendations are met by a PS, which is less palatable and varied than the wide-ranging natural protein sources in non-PKU diets. In this study, the prescribed intake of the PS (study product + baseline PS taken alongside study product) provided at least 80% of the recommended total protein prescriptions for the majority of the patients. Furthermore, the recommendations for the total protein intake in PKU are generally higher than those for non-PKU populations, because adjustments are necessary to compensate for the lower biological efficiency of AA-PSs and lower protein quality of the mainly vegetable-origin protein sources in this diet [20,21]. A large proportion of vitamin, mineral, and trace element intakes are usually provided by the PS, as the rest of the diet typically provides an insufficient intake of nutrients such as calcium, iron, zinc, selenium, vitamin B_12_, and vitamin D in quantities that would meet requirements [25,53]. Thus, if adherence to the PS prescription is below recommendations, the intake of a range of nutrients other than protein is compromised, which, in turn, may have an impact on clinical outcomes [29,37]. Though some encouraging reports have suggested that cGMP-PSs may improve the adherence of patients with PKU [38,39,41,42,43], only a limited number of studies have investigated the potential impact of the long-term usage of cGMP-PSs on nutritional status, mainly showing no statistically significant changes in biochemical data [50,54].

It is widely accepted that the L-AAs that provide the protein sources in AA-PSs are associated with a lower biological efficiency compared to natural protein sources [20]: the L-AAs in AA-PSs do not require digestion and are absorbed by the small intestine, whereas protein sources such as larger polypeptides and intact protein that require digestion are less rapidly absorbed [55], which, in turn, influences their utilization [56]. In PKU, there is some preliminary evidence that cGMP-PSs may slow the rate at which AAs are absorbed and improve nitrogen retention [39,42], and there has been speculation as to the potential physiological consequences of these differences and whether cGMP-PSs might potentially have an influence on outcomes such as growth, body composition, and bone health [40,50]. In a recent long-term study on children with PKU that investigated the growth outcomes and body composition in children fed either cGMP-PSs or AA-PSs, Daly et al. [57] reported a trend towards the cGMP group being taller, with an improved lean body mass and decreased fat mass, although the between-group differences for these outcomes did not reach statistical significance. Considering that cGMP-PSs are growing in popularity, the two new cGMP-PSs evaluated in our study add another choice for patients and the results provide valuable new data on the use of cGMP-PSs in the management of PKU. However, our study presents some limitations, including a small sample size, the lack of a control group, and the need for a broader evaluation of outcome measures (effects on appetite, nutritional intake, body composition, and clinical outcomes) in the short and longer term. Therefore, the full clinical potential of cGMP-PSs in the management of PKU requires further investigation.

## 5. Conclusions

As well as achieving protein and micronutrient intake from their PS, it is important that patients have a wide variety of palatable PSs that are available in a range of flavours, formats, and protein densities to minimise compliance fatigue with a lifelong and challenging dietary regimen. These new products, which were acceptable, well tolerated, and did not seem to adversely affect Phe control, will widen the choice of cGMP-PSs currently available for the management of children and adults with PKU.

## Figures and Tables

**Table 1:** Baseline characteristics of study population.

	Powder cGMP-PS (*n* = 13)	Liquid cGMP-PS (*n* = 10)
Gender	7 F, 6 M	4 F, 6 M
Age in years, mean (range)	37 (12–65)	24 (7–49)
BMI within ranges *:		
Normal, *n* (%)	4 (31%)	3 (30%)
Overweight, *n* (%)	5 (38%)	4 (40%)
Obese, *n* (%)	4 (31%)	2 (20%)
**PS at baseline:**		
cGMP-PS only, *n* (%)	7 (54%)	3 (30%)
Combination of cGMP-PS + AA-PS, *n* (%)	3 (23%)	3 (30%)
AA-PS only, *n* (%)	3 (23%)	4 (40%)
**Protein equivalent from baseline PS** in g/day (range)	61 (40–80)	65 (60–80)
**Phe control** ^**†**^**:**		
Within target, *n* (%)	7 (54%)	7 (70%)
Outside target, *n* (%)	4 (31%) ^‡^	3 (30%)
Fluctuating around target, *n* (%)	2 (15%)	none

* BMI ranges for adults: normal/healthy between 18.5–24.9; overweight between 25–29.9; and obese over 30. BMI range for children <18 years of age: age-specific score determined using the BMI centile charts produced by the Royal College of Paediatrics and Child Health. ^†^ Target treatment ranges for Phe control: 120–360 µmol/L for children <12 years and <600 µmol/L for children ≥12 years and adults. ^‡^ 3 subjects had Phe levels above upper limit of acceptable range and 1 subject had phe level below lower limit of acceptable range. Abbreviations: cGMP-PS, protein substitute with glycomacropeptide supplemented with several L-amino acids; AA-PS, amino-acid-based protein substitute; F, females; M, males; BMI, body mass index; Phe, phenylalanine; and n, number of patients.

**Table 2:** Combinations of protein substitutes used in the intervention period.

Study Products Combined with	Powdered cGMP-PS (*n*)	Liquid cGMP-PS (*n*)
Other cGMP-PS *	7	3
Other cGMP-PS * + AA-PS **	3	4
AA-PS **	3	3

* Other cGMP-PS included PKU Sphere 20 (Vitaflo), PKU Sphere 15 (Vitaflo), and PKU GMPower (Mevalia). ** AA-PS included PKU Air 20 (Vitaflo), PKU Air 15 (Vitaflo), Phlexy 10 tablets (Nutricia), PKU Cooler 20 (Vitaflo), PKU Cooler 15 (Vitaflo), and PKU Lophlex LQ 20 (Nutricia). *n*, number of patients.

**Table 3:** Recommended total protein prescription (natural protein + PS) and contributions from study products as well as all PS.

	Powdered cGMP-PS	Liquid cGMP-PS
Total protein prescription in g/d, mean (range) *	76 (55–92)	74 (66–88)
Percentage of total protein prescription from study product, mean (range) **	40 (22–98)	30 (23–51)
Percentage of total protein prescription from PS (all sources), mean (range) **	81% (52–93%)	88% (80–96%)
PS (all sources) intake provides ≥80% of total protein prescription, *n*	8	10
PS (all sources) intake provides 50–<80% of total protein prescription, *n*	5	0

* Total protein prescription was determined from Phe allowance (1 exchange = 50 mg Phe = 1 g protein) + PS prescription. Actual protein intakes from food sources may have differed. ** Patients replaced part of their baseline PS with the study products or took the study products in addition to their baseline PS to better meet their protein requirements. None of the patients took the study products as their sole PS. *n*, number of patients.

## Data Availability

The data presented in this study are available on request from the corresponding author.

## Appendix A

**Table A1:** Nutritional Information of the powdered and liquid cGMP-PS.

Average Contents (per 33.4 g and 250 mL Serving)		Powdered cGMP-PS	Liquid cGMP-PS
**Energy:**	kcal	98	99
	kJ	414	420
**Protein Equivalent:**	g	20	20.0
**Carbohydrate:**	g	4.5	3.2
sugars	g	2.8	0.15
**Fat:**	g	0.06	0.35
saturates	g	<0.03	0.07
monounsaturates	g	0	0.08
polyunsaturates	g	0	0.18
eicosapentaenoic acid (EPA)	mg		30.0
docosahexaenoic acid (DHA)	mg		120.0
**Fibre:**	g	0	2.10
**Minerals:**			
sodium	mg (mmol)	336 (16.1)	317 (13.8)
potassium	mg (mmol)	525 (13.4)	386 (9.87)
chloride	mg (mmol)	265 (7.4)	144.0 (4.06)
calcium	mg (mmol)	482 (12.0)	461 (11.5)
phosphorus	mg (mmol PO_4_)	473 (15.3)	384 (12.40)
magnesium	mg (mmol)	116 (4.8)	128.0 (5.27)
iron	mg	6.0	5.49
zinc	mg	4.7	4.75
copper	mg	0.5	0.58
manganese	mg	0.3	0.54
molybdenum	µg	24.4	21.0
selenium	µg	24.4	31.4
chromium	µg	17.0	16.70
iodine	µg	62.8	70.1
**Vitamins:**			
vitamin A	µg	184	287
vitamin D_3_	µg	9.0	10.0
vitamin E	mg α-TE	6.1	6.0
vitamin K_1_	µg	28.9	35.0
thiamin	mg	0.57	0.55
riboflavin	mg	0.73	0.60
niacin	mg (mg NE)	6.0 (14.4)	7.0 (15.30)
pantothenic acid	mg	2.0	3.0
vitamin B_6_	mg	0.67	0.65
folic acid	µg	80.2	100
vitamin B_12_	µg	1.4	1.67
biotin	µg	14.4	15.0
vitamin C	mg	28.9	31.0
**Amino Acids:**			
l-alanine	g	0.80	0.80
l-arginine	g	1.0	0.96
l-aspartic acid	g	1.2	1.18
l-cystine	g	0.57	0.58
l-glutamic acid	g	2.7	2.69
Glycine	g	0.14	0.14
l-histidine	g	0.80	0.78
l-isoleucine	g	1.5	1.45
l-leucine	g	3.0	3.00
l-lysine	g	0.86	0.86
l-methionine	g	0.27	0.27
l-phenylalanine	mg	36.0	36.0
l-proline	g	1.6	1.53
l-serine		1.0	1.02
l-threonine	g	2.3	2.25
l-tryptophan	g	0.47	0.50
l-tyrosine	g	2.0	2.00
l-valine	g	1.7	1.75
**Others:**			
l-carnitine	mg	26.5	28.5
Taurine	mg	60.1	60
choline	mg	120	163
inositol	mg	23.8	37.5
Osmolality	mOsm/kg H_2_O	530	390
Osmolarity	mOsmol/L	480	350
potential renal solute load	mOsmol/L	782	617

**Table A2:** Acceptability of the powdered cGMP-PS.

	Great	Good	Ok	Bad	Terrible
**Attribute**, *n* (%)					
Appearance, *n* = 13	1 (8)	6 (46)	5 (38)	1 (8)	0 (0)
Smell, *n* = 13	1 (8)	7 (54)	4 (30)	1 (8)	0 (0)
Taste, *n* = 13	1 (8)	3 (23)	6 (46)	3 (23)	0 (0)
Texture, *n* = 12	1 (8)	5 (42)	2 (17)	4 (33)	0 (0)
Ease of mixing, *n* = 13	3 (23)	2 (16)	3 (23)	3 (23)	2 (15)
Ease of taking, *n* = 13	3 (23)	2 (15)	6 (47)	2 (15)	0 (0)
Aftertaste, *n* = 13	2 (15)	2 (15)	6 (47)	3 (23)	0 (0)
Breath smell, *n* = 12	1 (8)	4 (33)	3 (26)	4 (33)	0 (0)
Overall acceptability, *n* = 13	1 (8)	4 (31)	5 (38)	3 (23)	0 (0)

**Table A3:** Acceptability of the liquid cGMP-PS.

	Great	Good	Ok	Bad	Terrible
**Attribute**, *n* (%)					
Appearance, *n* = 10	0 (0)	5 (50)	4 (40)	1 (10)	0 (0)
Smell, *n* = 10	1 (10)	4 (40)	3 (30)	0 (0)	2 (20)
Taste, *n* = 10	3 (30)	1 (10)	4 (40)	2 (20)	0 (0)
Texture, *n* = 10	2 (20)	3 (30)	5 (50)	0 (0)	0 (0)
Ease of taking, *n* = 10	4 (40)	3 (30)	2 (20)	1 (10)	0 (0)
Aftertaste, *n* = 9	2 (22)	1 (11)	4 (45)	2 (22)	0 (0)
Breath smell, *n* = 9	1 (11)	1 (11)	4 (45)	1 (11)	2 (22)
Tolerance, *n* = 10	3 (30)	5 (50)	1 (10)	1 (10)	0 (0)
Overall acceptability, *n* = 10	3 (30)	5 (50)	2 (20)	0 (0)	0 (0)
